# Registration of Panoramic/Fish-Eye Image Sequence and LiDAR Points Using Skyline Features

**DOI:** 10.3390/s18051651

**Published:** 2018-05-21

**Authors:** Ningning Zhu, Yonghong Jia, Shunping Ji

**Affiliations:** School of Remote Sensing and Information Engineering, Wuhan University, Wuhan 430079, China; jishunping@whu.edu.cn

**Keywords:** MMS LiDAR points, sequence of panorama/fish-eye images, skyline-based registration, brute force optimization

## Abstract

We propose utilizing a rigorous registration model and a skyline-based method for automatic registration of LiDAR points and a sequence of panoramic/fish-eye images in a mobile mapping system (MMS). This method can automatically optimize original registration parameters and avoid the use of manual interventions in control point-based registration methods. First, the rigorous registration model between the LiDAR points and the panoramic/fish-eye image was built. Second, skyline pixels from panoramic/fish-eye images and skyline points from the MMS’s LiDAR points were extracted, relying on the difference in the pixel values and the registration model, respectively. Third, a brute force optimization method was used to search for optimal matching parameters between skyline pixels and skyline points. In the experiments, the original registration method and the control point registration method were used to compare the accuracy of our method with a sequence of panoramic/fish-eye images. The result showed: (1) the panoramic/fish-eye image registration model is effective and can achieve high-precision registration of the image and the MMS’s LiDAR points; (2) the skyline-based registration method can automatically optimize the initial attitude parameters, realizing a high-precision registration of a panoramic/fish-eye image and the MMS’s LiDAR points; and (3) the attitude correction values of the sequences of panoramic/fish-eye images are different, and the values must be solved one by one.

## 1. Introduction

Motivated by applications of vehicle navigation, urban planning and autonomous driving, the need for 3D information on urban areas has dramatically increased in recent years. Mobile mapping systems (MMS) technology has been widely used for efficient data acquisition. Information obtained by an MMS mainly includes a sequence of optical images captured by a camera and point clouds obtained by LiDAR. Optical images have rich texture information, and point clouds mainly reflect the spatial characteristics. Registering images and point clouds has important theoretical and practical value.

Optical image (2D) and point cloud (3D) data are two types of data. The imaging model of an optical image is a collinear equation. The point cloud reflects the position of an object by transmitting a laser pulse. Thus, the geometric reference frame of LiDAR points and an optical image is different. There are 3 key problems that must be solved before registration: the primitive pairs, the registration model and parameter optimization. To capture large scenes in a single image, a panoramic camera could be used on an MMS. The most common type of panoramic camera, such as Ladybug, consists of a multiple fish-eye camera rig. Images with a narrow field are first obtained by fish-eye lenses, and then projected and resampled to stitch together a panoramic image. The acquisition efficiency of an MMS camera can reach 10 fps/s; assuming the speed of the vehicle is 72 km/h, the image density is up to 2 m. As a result, the registration method of the panorama/fish-eye image and the LiDAR points should be automated and efficient.

The existing algorithms for registering panoramic/fish-eye images and LiDAR points mostly rely on control points (control point-based registration method) or GPS/IMU information (original registration method) [1,2]. LiDAR points and panoramic/fish-eye images can be registered well by control points; however, automatic extraction of the control point is a difficult task at present. Furthermore, it is impossible to extract the control point from the LiDAR points and the sequence of images manually. Original registration method uses the position and attitude parameters obtained by GPS/IMU directly, however, the accuracy of GPS/IMU is affected by interference, such as dropouts. This error accumulates over time and may cause unreliable registration for long sequence images; thus, the precision of original registration method is low. Wang et al. [3] proposed an automatic registration of mobile LiDAR and spherical panoramas; however, only part of the panoramic image was used, and the registration was based on a conventional frame camera model. Li et al. [2] proposed an automatic registration method based on semantic features extracted from panoramic images and point clouds, but the accuracy of this method relies on the extraction of primitive pairs (parked vehicles) and is only suitable for urban scenes.

A linear feature exceeds a point feature regarding registration accuracy; linear features are more reliably, accurately, and automatically extracted from images and LiDAR points [1,4]. The skyline is easily extracted from LiDAR points and optical images. The skyline registration method aims at retrieving the camera’s pose via skyline points/pixels matching. Hofmann et al. [5] extracted the building skyline from real and synthetic images; both skylines are then merged using the ICP method. However, their approach did not analyze special conditions of the skyline, such as missing data, jagged skylines, or even too much vegetation; moreover, it uses a frame camera rather than a panoramic/fish-eye image in an MMS. At present, registration methods of point clouds and optical images can be divided into 3 categories: 2D-2D-based methods, 2D-3D-based methods and 3D-3D-based methods.

**2D-2D-based methods.** 2D-2D-based methods cast the problem of image to point cloud registration as an iterative process of image to image registration. The point cloud should be turned into a synthetic image on which the real image can be registered, and then, initial registration parameters can be estimated using both images. The synthetic image is regenerated using the new parameters, and then, this process is iterated. 2D-2D-based methods can be divided into feature-based and statistical analysis.

Feature-based registration methods rely on feature points/lines obtained by SIFT [6], SURF [7], and ASIFT [8] on real and synthetic images, followed by establishment of the correspondences through common features to realize registration [9,10,11,12,13,14]. Statistical analysis-based registration methods are widespread for aligning an image to another image [15]; mutual information (MI) proposed by Viola [16] is the most commonly used statistical method. MI measures the similarity between two images based on the dependency of the intensity distribution [17,18,19,20]. Taylor and Nieto [21] proposed a modified form of MI using particle swarm optimization; Pascoe et al. [22] introduced a normalized information distance metric based on MI and entropy variation to retrieve the camera position.

**2D-3D-based methods.** 2D-3D-based methods rely on identifying the same point/line from the image and the LiDAR points and then constructing a strict geometric model to achieve high-precision registration. This method uses the correspondence between 2D and 3D points/line; the camera pose is finally obtained by solving a perspective-n-point (PnP) problem using the EPnP algorithm [23,24]. The principle and concept of line-based registration, especially straight lines, were regarded as an extension of traditional point features. Zhang [25] proposed the concept of “generalized points”, which is a series of connected points representing a linear feature. According to this concept, the traditional collinearity model can accommodate more complicated features, such as circles and rectangles, rather than only straight lines. Stamos et al. [26] and Schenk [27] laid a solid foundation in line feature-based registration, and Liu et al. [28,29] utilized linear features to determine the camera orientation and position relative to the 3D model.

**3D-3D**-based **methods.** 3D-3D-based methods require a sequence of images for 3D reconstruction and then 3D matching to achieve registration. Structure from motion (SFM) is a widely used method that reconstructs 3D points using a set of images [30,31]. A 3D matching algorithm, such as iterative closest point (ICP) [32] and normal distributions transform (NDT) [33], is often used for point registration. Zheng et al. [34] used bundle adjustment for a sequence of images; the points obtained by adjustment were matched with the laser point cloud by ICP. Zhao et al. [35] used stereo vision technology to process 3D reconstruction and used the ICP algorithm to achieve 3D point cloud registration. Abayowa et al. [36] presented a coarse to fine strategy in the estimation of the registration parameters without initial alignment.

**Figure 1:**
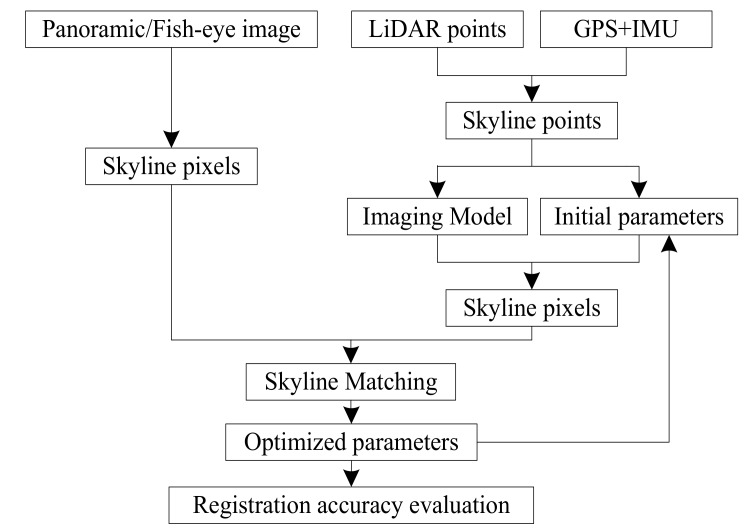
Flow chart of the skyline-based registration method.

We propose utilizing a rigorous imaging model and skyline-based method for automatic registration of LiDAR points and a sequence of panoramic/fish-eye images. This method improves the accuracy of registration automatically through optimizing attitude parameters. Figure 1 shows the flow chart of this method. This paper is organized as follows. Section 2 provides the rigorous panoramic/fish-eye image registration model and the skyline matching method. In Section 3, conducted experiments that verify the effectiveness of the skyline-based method are described. Section 4 discusses the parameters in skyline pixels/points matching and the precision/automation of our method. Finally, conclusions are presented in Section 5.

## 2. Materials and Methods

### 2.1. Materials

The MMS used in this paper was jointly developed by Wuhan University and Leador Spatial Information Technology Corporation, configured with a panoramic camera, three low-cost SICK laser scanners (one for ground and two for facades) and a GPS/IMU [1], they are connected by a precision mechanical device with accurate calibration. The panoramic camera is a Ladybug5 (FLIR Integrated Imaging Solutions Inc., Richmond, BC, Canada), which includes 6 high-definition Sony ICX655 CCDs, with 5 in the side (horizontal) and 1 at the top (vertical). The camera can perform image acquisition, processing, correction and stitching of a panoramic image in real-time.

**Figure 2:**
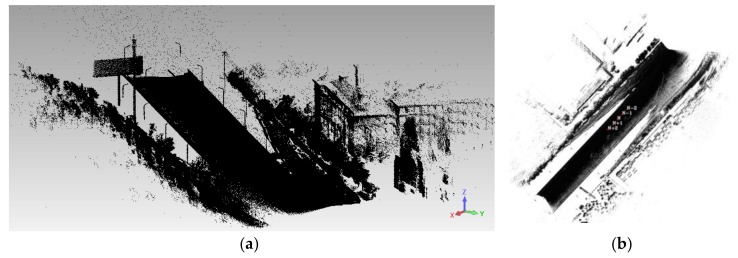
LiDAR points data. (**a**) Main view of LiDAR points. (**b**) Plane map of LiDAR points.

**Figure 3:**
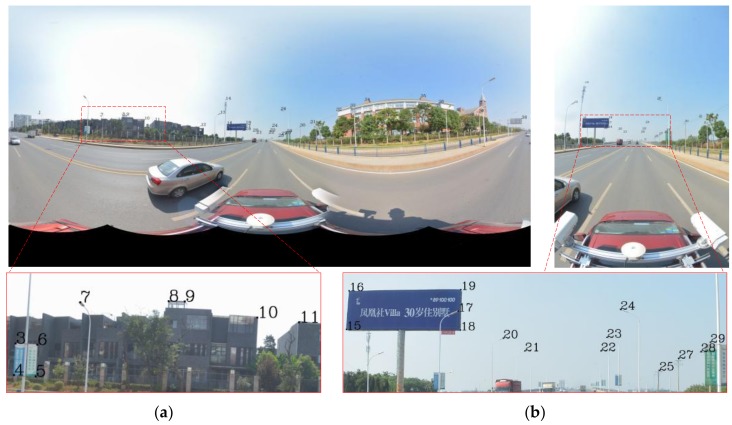
Panoramic/fish-eye image and control points distribution. (**a**) Panoramic image (4000 × 8000). (**b**) Fish-eye image (6000 × 4000).

**Figure 4:**
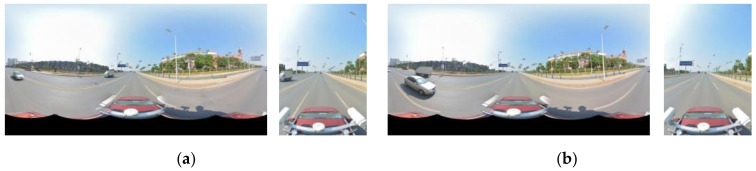

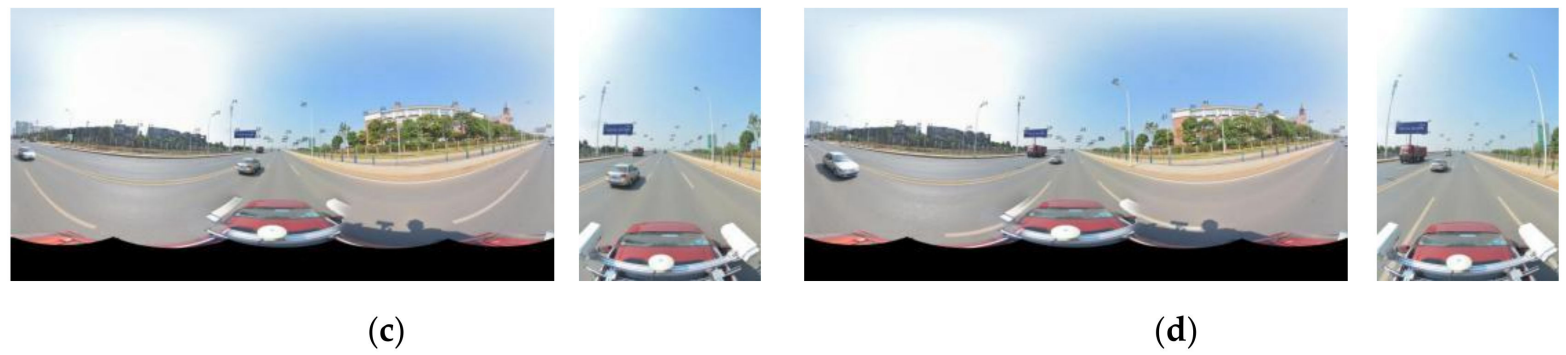
Sequence of panoramic/fish-eye images. (**a**) *N* − 2; (**b**) *N* − 1; (**c**) *N* + 1; (**d**) *N* + 2.

Figure 2 and Figure 3 show the point data and the optical image of the MMS, respectively. The image data include a fish-eye image (6000 × 4000) along the road direction and a panoramic image (4000 × 8000) stitched by 6 fish-eye images. The point data of the MMS corresponds to the image at the same place; this road has a length that is approximately 220 m, the data of which includes 3 million points. In addition, the initial values of the imaging position and attitude, as well as the calibration information of the MMS sensors, are known. Based on the current panoramic/fish-eye image (*N*), 4 panoramic/fish-eye images were selected on both sides of *N*, which constituted 5 consecutive images (*N* − 2, *N* − 1, *N*, *N* + 1, and *N* + 2). This sequence of panoramic/fish-eye images is shown in Figure 4.

To compare the accuracy of different registration methods, 38 control points are selected manually; these points are mainly distributed on the corners of buildings and billboards, the tops of lamps, etc. As shown in Figure 1 and Figure 3, the panoramic and fish-eye images contains 38 and 19 control points, respectively; an exception is that only 17 control points are in the fish-eye image (*N* + 2). In Appendix A, Table A1 lists the 3D coordinates of the 38 control points obtained from the LiDAR points; Table A2 lists the 2D image coordinates of the 38 control points in the panoramic image; and Table A3 lists the 2D image coordinates of control points in the fish-eye image.

### 2.2. Panoramic/Fish-Eye Image Registration Model 

The imaging mode of a panoramic/fish-eye image must be known before registration. The panoramic camera used in an MMS is composed of multiple fish-eye lenses at present; the fish-eye images can be stitched together to form a panoramic image according to a fixed model. As shown in Equation (1), the collinear equation is a classic imaging model. The *Y* axis is perpendicular to the image plane (XOZ) in this model, and the panoramic/fish-eye imaging model can be transformed by a collinear equation.

(1) $$\left\{ \begin{array}{l} r=-f\cdot \bar{Z}/\bar{Y}\\ c=-f\cdot \bar{X}/\bar{Y} \end{array}\right.$$

where ${\left[\bar{X}\;\bar{Y}\;\bar{Z}\right]}^{T}=R\cdot {\left[\bar{x}\;\bar{y}\;\bar{z}\right]}^{T}$, $R=R_{X}\cdot R_{Y}\cdot R_{Z}$, $\left[\bar{x}\;\;\bar{y}\;\;\bar{z}\right]=[x-X_{S}\;y-Y_{S}\;z-Z_{S}]$,

$$R_{X}=\left[\begin{array}{l} 1\;\;\;\;\;\;\;\;\;0\;\;\;\;\;\;\;\;\;\;\;0\\ 0\;\;\;\cos(r_{x})\;\;-\sin(r_{x})\\ 0\;\;\;\sin(r_{x})\;\;\;\cos(r_{x})\;\; \end{array}\right]R_{Y}=\left[\begin{array}{l} \cos(r_{y})\;\;\;0\;\;\;-\sin(r_{y})\;\\ \;\;\;\;0\;\;\;\;\;\;\;1\;\;\;\;\;\;\;0\\ \sin(r_{y})\;\;\;\;0\;\;\;\cos(r_{y})\;\; \end{array}\right]R_{Z}=\left[\begin{array}{l} \cos(r_{z})\;\;-\sin(r_{z})\;\;0\\ \sin(r_{z})\;\;\;\cos(r_{z})\;\;\;\;0\\ \;\;\;0\;\;\;\;\;\;\;\;\;\;\;0\;\;\;\;\;\;\;1 \end{array}\right]$$

(*x y z*) is the coordinate of an object, *f* is the imaging lens focal length, (*X_S_ Y_S_ Z_S_*) is the imaging position, (*r_x_ r_y_ r_z_*) is the imaging attitude, and (*r c*) is the perspective projection coordinate.

#### 2.2.1. Panoramic/Fish-Eye Imaging Model

There are 4 types of fish-eye lens projections: equidistant projection, orthographic projection, equisolid-angle projection and stereographic projection [37,38]. The panoramic stitching models include a spherical model, cylinder model and cube model. Considering the experimental data in our paper, an equidistant projection and a spherical model were used as examples.

The equidistant projection of a fish-eye lens can be expressed as:

(2) $$\left\{ \begin{array}{l} r\prime =-f\cdot \bar{Z}/\bar{Y}\cdot \theta /\tan\theta \\ c\prime =-f\cdot \bar{X}/\bar{Y}\cdot \theta /\tan\theta \end{array}\right.$$

where $\theta =a\tan(\sqrt{{\bar{Z}}^{2}+{\bar{X}}^{2}}/\bar{Y})$, and (*r*′ *c*′) is the coordinate of the object in the fish-eye image.

As shown in Equation (3), for a panoramic image using the spherical model, take vertical angle (*v*) as the row coordinate and the horizontal angle (*h*) as the column coordinate. Considering h∈[−π π] and v∈[−π/2 π/2], the size of the panoramic image column is 2 times that of a row.

(3) $$\left\{ \begin{array}{l} \tan\;v=\bar{Z}/\sqrt{{\bar{X}}^{2}+{\bar{Y}}^{2}}\\ \tan\;h=\bar{X}/\bar{Y} \end{array}\right.$$

The imaging parameters can be obtained accurately by the control points. Equations (4) and (5) are transformed by Equations (2) and (3) and represent the computational model of the panoramic/fish-eye image.

(4) $$\left\{ \begin{array}{l} \bar{Z}/\bar{Y}=-r\prime \cdot \tan\theta /r_{d}\\ \bar{X}/\bar{Y}=-c\prime \cdot \tan\theta /r_{d} \end{array}\right.$$

where $r_{d}=\sqrt{r\prime^{2}+c\prime^{2}}$, $\theta =r_{d}/f$, and (*r*′ *c*′) is the fish-eye image coordinate.

(5) $$\left\{ \begin{array}{l} \bar{Z}/\bar{Y}=\tan\;v\cdot \sqrt{1+\tan^{2}h}\\ \bar{X}/\bar{Y}=\tan\;h \end{array}\right.$$

where v=r′′/row⋅π, h=c′′/col⋅2π, (*row col*) expresses the size of the panoramic image, and (*r*″ *c*″) is the panoramic coordinate.

Let the right side of Equations (4) and (5) equal *p_1_* and *p_2_*, which are known as the image coordinate. The adjustment model of a panoramic/fish-eye image is shown in Equation (6); these variables can be calculated by the DLT method.

(6) $$\left\{ \begin{array}{l} p_{1}=\frac{R_{20}\cdot \;\bar{x}+R_{21}\cdot \;\bar{y}+\;R_{22}\cdot \;\bar{z}}{R_{10}\cdot \;\bar{x}+R_{11}\cdot \;\bar{y}+\;R_{12}\cdot \;\bar{z}}\\ p_{2}=\frac{R_{00}\cdot \;\bar{x}+R_{01}\cdot \;\bar{y}+\;R_{02}\cdot \;\bar{z}}{R_{10}\cdot \;\bar{x}+R_{11}\cdot \;\bar{y}+\;R_{12}\cdot \;\bar{z}} \end{array}\right.$$

#### 2.2.2. Registration Accuracy Evaluation

Substituting the solved variables and control points’ coordinates into Equation (6), *p_1_* and *p_2_* can be calculated, and then the panoramic/fish-eye image coordinates of control points can be obtained by Equations (7) and (8).

(7) $$\left\{ \begin{array}{l} v=a\tan(p_{1}/\sqrt{1+p_{2}{}^{2}})\\ h=a\tan(p_{2}) \end{array}\right.$$

(8) $$\left\{ \begin{array}{l} r\prime =-f\cdot p_{1}\cdot \theta /\tan\theta \\ c\prime =-f\cdot p_{2}\cdot \theta /\tan\theta \end{array}\right.$$

where $\theta =a\tan(\sqrt{p_{1}{}^{2}+p_{2}{}^{2}})$, and the other parameters are the same as those previously indicated.

The registration error (δ) is given by Equation (9) and is considered to be the precision index of registration.

(9) $$\delta =\sqrt{\sum_{i=1}^{m}\left[{\left(r_{i}-r_{i}\prime \right)}^{2}+{\left(c_{i}-c_{i}\prime \right)}^{2}\right]/m}$$

where *m* is the number of control points, (*r_i_ c_i_*) expresses the image coordinate of the control points from Equations (7) and (8), and (*r_i_*′ *c_i_*′) expresses the real coordinate of the control points.

### 2.3. Skyline-Based Method

The skyline-based method includes skyline pixels extracted from a panoramic/fish-eye image, skyline points extracted from LiDAR points and skyline pixels/points matching.

#### 2.3.1. Skyline Pixels Extracted from Panoramic/Fish-Eye Image

**Figure 5:**
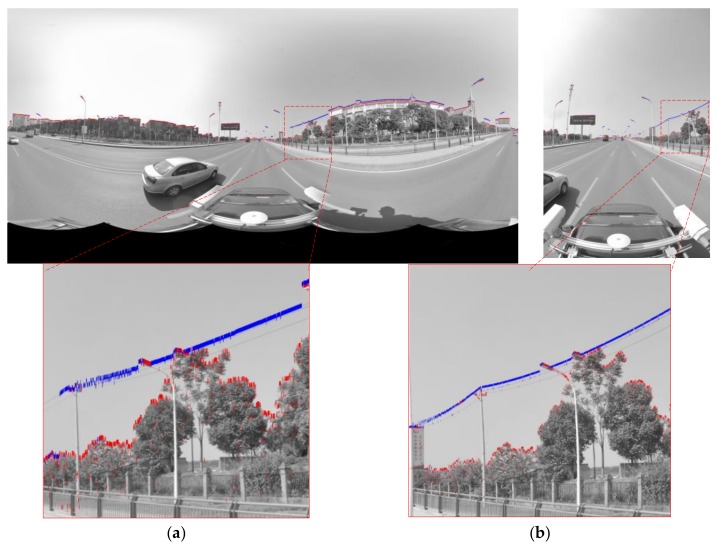
Skyline pixels (display in bold). (**a**) Panoramic image skyline pixels (red pixels) optimization. (**b**) Fish-eye image skyline pixels (red pixels) optimization.

Because the difference in the image pixel value between the sky and other objects is significant, the skyline can be easily extracted by this feature. The first grey jump as the skyline pixels are searched from above to below and column by column, the skyline pixels (blue pixels) extracted from the panoramic/fish-eye image can be obtained, as shown in Figure 5a,b, which include the top profile of buildings, trees, billboards, power lines and street lamps. Considering the linear feature of a power line and the discrete characteristics of LiDAR points, power line points extracted from LiDAR points are incomplete; thus, power line pixel interference must be eliminated by setting a jump buffer. Figure 5a,b show skyline pixels (red pixels) after optimization in which the power line pixels have been eliminated.

#### 2.3.2. Skyline Points Extracted from LiDAR Points

Skyline points extracted from LiDAR points are influenced by imaging position, imaging attitude, imaging focal length and imaging model. In particular, panoramic image registration is related to imaging position and attitude, and fish-eye image registration is related to imaging position, attitude and focal length. Imaging position and attitude can be obtained by GPS/IMU and calibration of the MMS, and imaging focal length is obtained by camera calibration. Next, skyline points can be extracted from LiDAR points as follows. First, generate a panoramic image with all points using Equation (3); the highest pixel in the image is calculated column by column, and the point corresponding to this pixel is a skyline point. The extraction algorithm of fish-eye image registration is similar; the skyline points include a large amount of noise, and no skyline points correspond to skyline pixels in some regions; therefore, constraints must be added in skyline matching to ensure the rigorous correspondence of skyline points and pixels.

#### 2.3.3. Skyline Pixels/Points Matching

The skyline pixels/points are extracted from the panoramic/fish-eye image and LiDAR points above. We define the image skyline pixels as $\Phi {\left\{r_{i}\;,c_{i}\right\}}_{i=0,1,\ldots m}$ and the skyline points as $\Omega {\left\{x_{i}\;,y_{i}\;,z_{i}\right\}}_{i=0,1,\ldots n}$ in this section to analyze the skyline matching method. The key of this method is to restore the correspondence between Φ and Ω. We solve this problem using the brute force optimization method. The position parameters have little influence on the image compared to the attitude parameters; therefore, we only optimize attitude parameters in this paper. As shown in Equation (10), the procedure follows from constructing the correcting matrix ***R***′, which consists of 3 correction angles (*r_x_*′ *r_y_*′ *r_z_*′), and then placing ***R***′ before ***R*** in the imaging model. (*r_x_*′ *r_y_*′ *r_z_*′) can be obtained by brute force optimization, and the registration of the LiDAR points and the image is performed when the skyline pixels/points are matched.

(10) $${\left[\bar{X}\;\bar{Y}\;\bar{Z}\right]}^{T}=R\prime \cdot R\cdot {\left[\bar{x}\;\bar{y}\;\bar{z}\right]}^{T}$$

where $R\prime =R_{X}\prime \cdot R_{Y}\prime \cdot R_{Z}\prime$,

$$R_{X}\prime =\left[\begin{array}{l} 1\;\;\;\;\;\;\;\;0\;\;\;\;\;\;\;\;\;\;0\\ 0\;\;\;\cos(r_{x}\prime )\;\;-\sin(r_{x}\prime )\\ 0\;\;\;\sin(r_{x}\prime )\;\;\;\;\cos(r_{x}\prime ) \end{array}\right]R_{Y}\prime =\left[\begin{array}{l} \cos(r_{y}\prime )\;\;\;0\;\;-\sin(r_{y}\prime )\\ \;\;\;\;0\;\;\;\;\;\;\;1\;\;\;\;\;\;\;0\\ \sin(r_{y}\prime )\;\;\;0\;\;\;\;\cos(r_{y}\prime ) \end{array}\right]R_{Z}\prime =\left[\begin{array}{l} \cos(r_{z}\prime )\;\;-\sin(r_{z}\prime )\;\;\;0\\ \sin(r_{z}\prime )\;\;\;\cos(r_{z}\prime )\;\;\;\;0\\ \;\;\;0\;\;\;\;\;\;\;\;\;\;\;0\;\;\;\;\;\;\;\;\;1 \end{array}\right]$$

First, the imaging position (*X_S_ Y_S_ Z_S_*), the attitude (*r_x_ r_y_ r_z_*) and the focal length (*f*) are obtained from GPS/IMU and calibration of the MMS [1] (as this calculation is very common, it is not described in detail here). Taking the maximum error of (*r_x_ r_y_ r_z_*) as the initial value range (*w*) and dividing it into *t* parts, as shown in Equation (11), (*t* + 1)^3^ panoramic/fish-eye synthetic images $\Omega \{r_{i}\;,c_{i}\}$ are generated using $\Omega \{x_{i}\;,y_{i}\;,z_{i}\}$ according to Equations (2), (3) and (10). Second, we calculate the similarity between $\Omega \{r_{i}\;,c_{i}\}$ and $\Phi \{r_{j}\;,c_{j}\}$, as shown in Equation (12). With the number of matching skyline pixels (*n*) used as the evaluation index, the highest similarity can be obtained from (*t* + 1)^3^ synthetic images, and taking the corresponding parameters (*i*, *j*, *k*) as the initial value of the next iteration, each iteration reduces the range by half.

(11) $$\left\{ \begin{array}{l} r_{x}^{,}{}_{\left(s+1\right)}=r_{x}^{,}{}_{\left(s\right)}-w_{\left(s\right)}+i\cdot 2\cdot w_{\left(s\right)}/t\\ r_{y}^{,}{}_{\left(s+1\right)}=r_{y}^{,}{}_{\left(s\right)}-w_{\left(s\right)}+j\cdot 2\cdot w_{\left(s\right)}/t\\ r_{z}^{,}{}_{\left(s+1\right)}=r_{z}^{,}{}_{\left(s\right)}-w_{\left(s\right)}+k\cdot 2\cdot w_{\left(s\right)}/t \end{array}\right.\;$$

where *s* expresses the *s*-th iteration, *w*_(*s*)_ *= w*/2*^s^*, *r*′*_x_*_ (0)_ *= r*′*_y_*_(0)_ *= r′_z_*_(0)_ = 0° and *i, j, k* = 0, 1, …, *t*.

(12) $$n=\left\{ \begin{array}{l} n++,\;\;\left|\Omega \{r_{c}\}-\Phi \{r_{c}\}\right|<e\\ n,\;\;\;\;\;\;\left|\Omega \{r_{c}\}-\Phi \{r_{c}\}\right||>e \end{array}\right.\;(c=1,\;2\;\ldots \;col)$$

where *e* denotes the gross error elimination threshold; when the row difference between $\Omega \{r_{i}\;,c_{i}\}$ and $\Phi \{r_{i}\;,c_{i}\}$ in the same column are less than *e*, *n* is added; otherwise they are unchanged. *n* reflects the skyline similarity between a real image and a synthetic image. *e* is used to eliminate mismatching of skyline pixels. Considering the differences of acquisition sensors, skyline points are impossible to match completely. For example, the building skyline in the left part of the panoramic image is easy to extract from the image; however, LiDAR points are missing in the corresponding region, and there is no matching skyline. *e* can eliminate this part of the skyline, such that it is not involved in the parameter optimization.

## 3. Experiments and Analysis

### 3.1. Comparison of the Registration Methods

**Figure 6:**
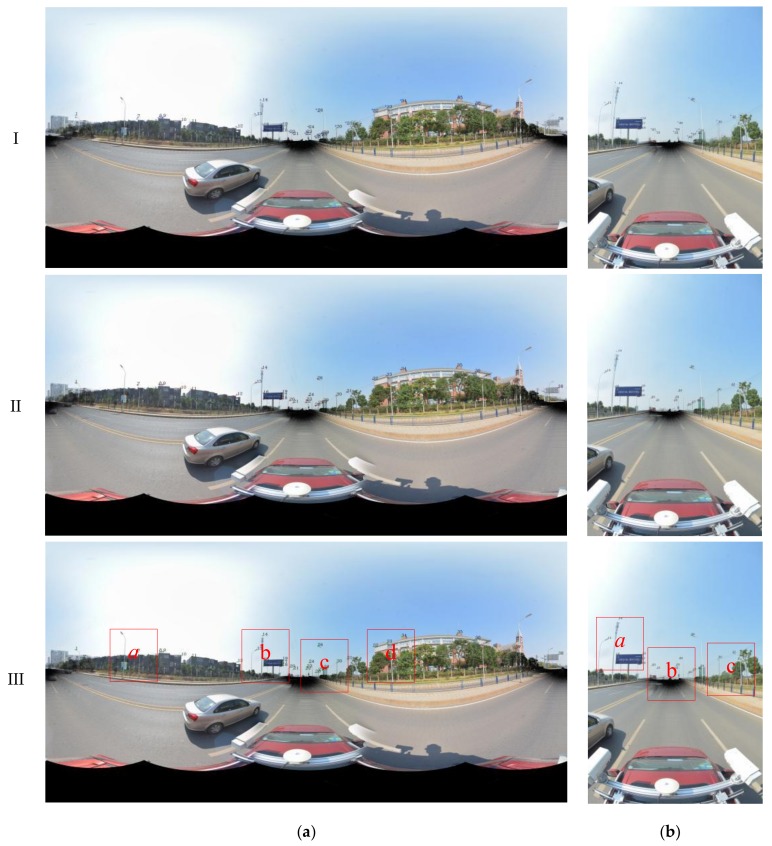
Registration result with 3 methods. (**a**) Panoramic image registration; (**b**) Fish-eye image registration.

To compare the accuracy of our method, the original registration method, the skyline-based registration method and the control point registration method (referred to below as method I, II and III, respectively) were used for the panoramic/fish-eye image registration. First, the original registration (I) was conducted according to Equations (1)–(3), and the registration parameters (*f*,*X_S_*,*Y_S_*,*Z_S_*,*r_x_*,*r_y_*,*r_z_*) were obtained by GPS/IMU and MMS calibration. Second, by taking advantage of control points for registration (III) according to Equations (4) and (5), this method does not require the initial value. The registration results of the panoramic/fish-eye image (*N*) with method I and III are shown in Figure 6.

**Figure 7:**
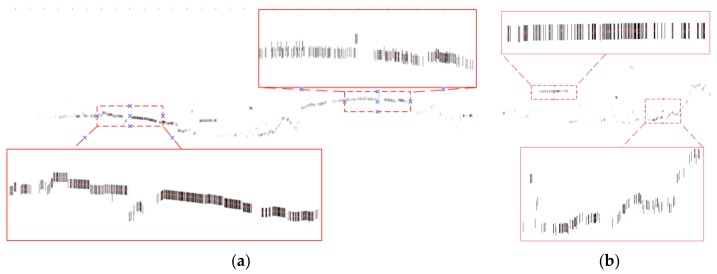
Matching skyline pixels, black pixels express real image skyline, display with 10 pixels width, red pixels express synthetic image skyline. (**a**) Panoramic image skyline (*n* = 1420); (**b**) Fish-eye image skyline (*n* = 454).

**Figure 8:**
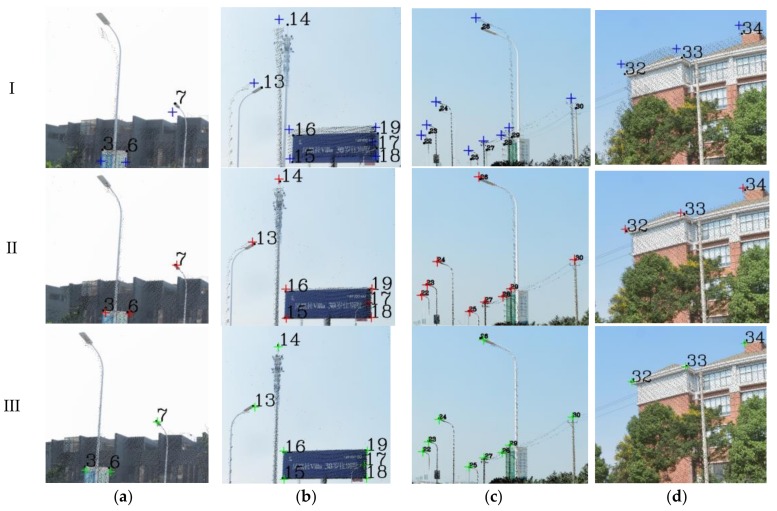
Local effect comparison of panoramic image registration, black squares express real location of control points; blue, red, and green crosses are synthetic location of control points calculated by method I, II, and III respectively. (**a**) Building and Lamp; (**b**) Lamp and Billboard; (**c**) Lamp; (**d**) Building and Tree.

**Figure 9:**
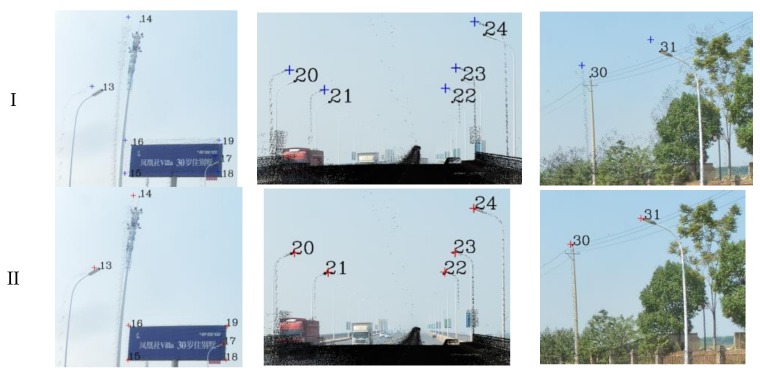

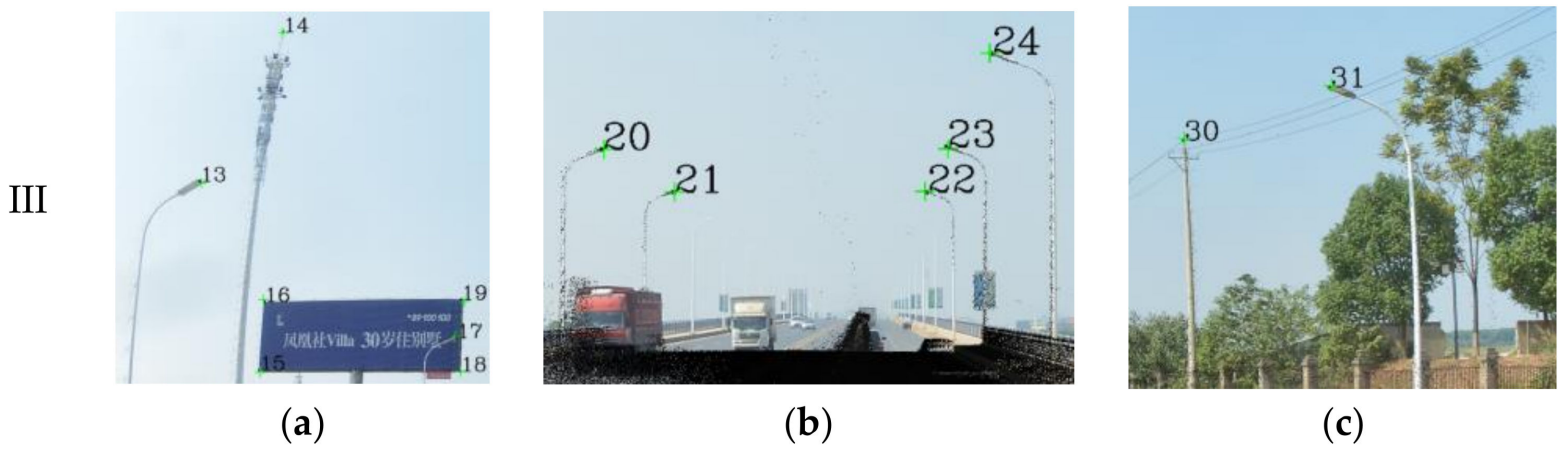
Local effect comparison of fish-eye image registration, black squares express real location of control points; blue, red, and green crosses are synthetic location of control points calculated by method I, II, and III respectively. (**a**) Lamp and Billboard; (**b**) Lamp; (**c**) Lamp and Tree.

The key parameters involved in the skyline registration method include: angle error range (*w*), parameter segmentation (*t*), gross error threshold (*e*), number of matching pixels (*n*) and registration error (*δ*). *n* is the evaluation index of the parameter optimization, *δ* expresses the matching error of 38 (19) control points. Herein, let *w* = 5°, *t* = 6, and *e* = 5 pixels. Next, the skyline is extracted from the panoramic/fish-eye image and the LiDAR points, as described above. The skyline points are used for imaging 7^3^ times by Equations (2), (3) and (10), and *n* between each generated image and real image skyline is calculated. We take both sides of maximum *n* as the starting value of the next iteration, and *w* is reduced by half in each iteration. After 6 iterations, the matching skyline pixels of the panoramic/fish-eye image are shown in Figure 7. The registration results with 3 methods are shown in Figure 6. Figure 8 and Figure 9 show local images to compare the registration effect.

As shown in Figure 7, the matching skyline pixels includes the top outlines of buildings, trees, lamps, etc. The influence of the unmatched skyline’s registration effect has been eliminated, such as the missing parts (the building in the left panoramic image) and cables. Simultaneously, considering the discrete types of point clouds and the continuity of the image, the number of matching skyline pixels(*n*) is far less than the column of the panoramic/fish-eye image. The proportion of the panoramic and fish-eye images is 17.75% and 11.35%, respectively. Figure 8 and Figure 9 show the local registration effect of the panoramic and fish-eye images, respectively, with 3 methods. A large dislocation is observed with method I, especially in the billboards, street lights, trees, buildings, etc. Conversely, method III can obtain the ideal effect, and all objects have a very high coincidence. The proposed skyline-based method can also achieve good registration, whose accuracy is between that of method I and that of method III. To quantitatively analyze the effect of the 3 methods, the registration error (*δ*) is calculated by the control points according to Equation (9). Table 1 lists *δ* in each iteration using our method, and Table 2 lists *δ* for 3 methods.

**Figure 10:**
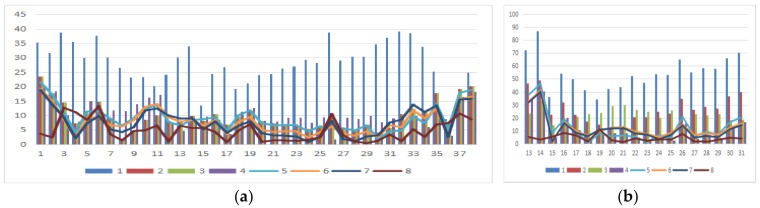
The error of 38/19 control points in the panoramic/fish-eye image with 3 methods, series 1 expresses the error of each control point in method I; similarly, series 2–7 express 6 iterations of our method, and series 8 expresses the result of method III. (**a**) Panoramic image; (**b**) Fish-eye image.

**Figure 11:**
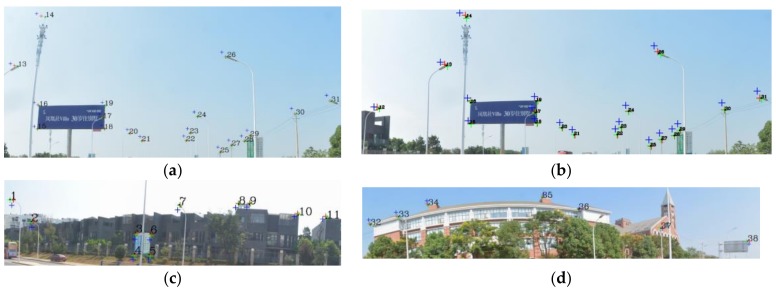
Control points displayed in the panoramic/fish-eye image. (**a**) Fish-eye image (*No.*13–31); (**b**) Panoramic image (*No.*12–31); (**c**) Panoramic image (*No.*1–11); (**d**) Panoramic image (*No.*32–38).

As shown in Table 1, *δ* becomes stable in both panoramic/fish-eye images after 6 iterations; *w* has been reduced to 0.15°, and the angle parameters are no longer the main factors affecting registration accuracy. Analysis of each iteration reveals that *δ* noticeably decreases as *n* increases, and that the rotation angle parameter has been optimized gradually. Table 2 lists *δ* of 3 methods. Our method has an accuracy (*δ*/image diagonal) of 0.97‰ and 2.04‰ in the panoramic and fish-eye images, respectively, which is greater than that of method III and less than that of method I. This conclusion is the same as the above image analysis. Finally, the position error of each control point with the 3 methods is analyzed in detail. Figure 10 shows the error of 38/19 control points in the panoramic/fish-eye image. Figure 11 shows the locations of all control points after registration.

According to Figure 10a,b, the error of each control point is clearly reduced after each iteration compared with method I, especially in the first iteration. Afterwards, the error maintenance reduces in subsequent iterations and gradually approaches that of method III. Figure 11 displays all control points calculated by the 3 methods on panorama/fish-eye images. We can see that the direction of the control points’ offset is consistent compared with the real position in method I, and this error is regular, indicating the optimized rotation angle has a theoretical basis.

### 3.2. Point Cloud Colouring

**Figure 12:**
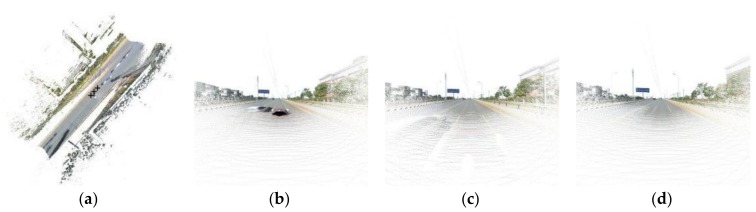
Texture point cloud imaging. (**a**) Plane map of texture points and the location of *N* − 1*, N, N* + 1(black crosses). (**b**–**d**) Perspective imaging of texture points in different location, (**b**) *N* − 1; (**c**) *N*; (**d**) *N* + 1.

LiDAR points can be given an RGB value after registration, so the points not only contain spatial information but also have texture information. Using texture points can achieve a texture image with any position and attitude. The procedure follows by setting the current imaging position as *N*, *N −* 1 and *N +* 1 to express the imaging positions before and after *N* in the sequence images, as shown in Figure 12a. We generate an image according to the perspective imaging model. The main axis is along the road direction and the other rotation angle is 0°. The imaging focal length is 250 pixels and the imaging size is 1000 × 1000. Figure 12b–d show the imaging results. Through texture points imaging, we can obtain the scene from an arbitrary location and attitude to meet different vision requirements.

### 3.3. Sequence of Panoramic/Fish-Eye Image Registration

**Figure 13:**
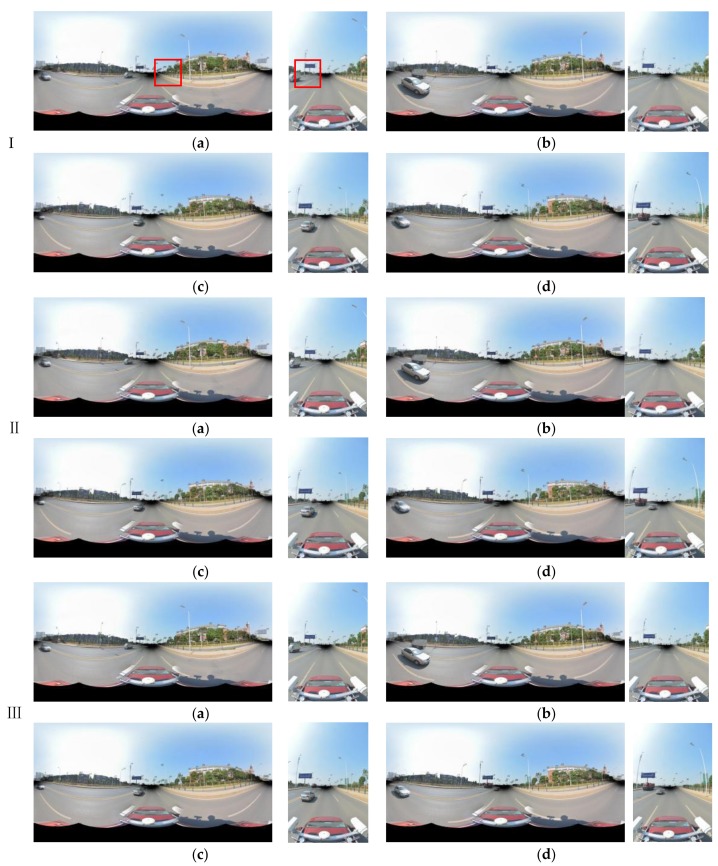
Registration result of panoramic/fish-eye image sequence with 3 methods. (**a**) *N* − 2; (**b**) *N* − 1; (**c**) *N* + 1; (**d**) *N* + 2.

**Figure 14:**
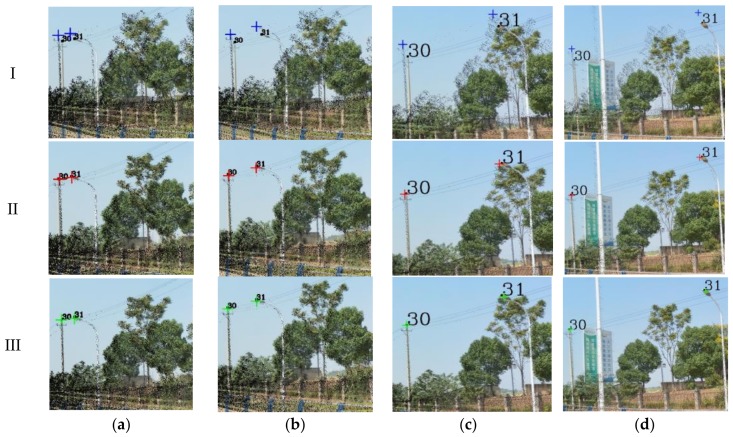
Local registration effect of panoramic images sequence with 3 methods. (**a**) *N* − 2; (**b**) *N* − 1; (**c**) *N* + 1; (**d**) *N* + 2.

**Figure 15:**
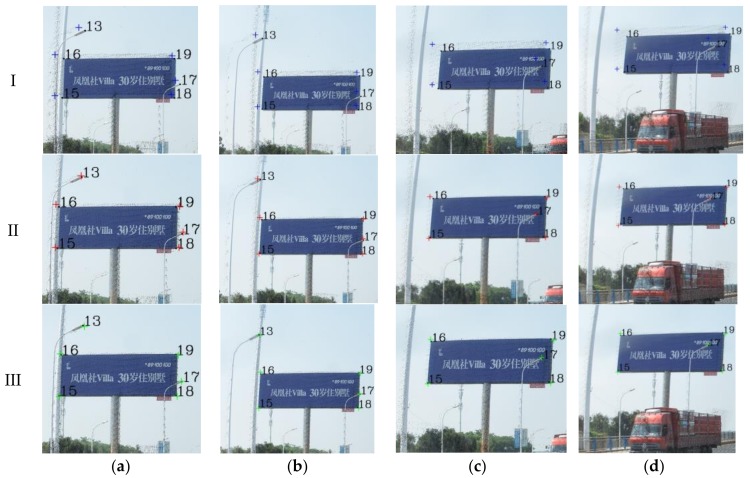
Local registration effect of fish-eye images sequence with 3 methods. (**a**) *N* − 2; (**b**) *N* − 1; (**c**) *N* + 1; (**d**) *N* + 2.

Our registration method is verified by the previous experiment. In this section, experiments are performed using our method with a sequence of panoramic/fish-eye images (*N* − 2, *N −* 1, *N* + 1, *N* + 2) to analyze the regularity of the optimized parameters. The parameters involved in our method are the same as those previously indicated. The rotation angle correction value (*r_x_*′ *r_y_*′ *r_z_*′) of each panoramic/fish-eye image can be obtained automatically. Figure 13 shows the registration result using the 3 methods. Figure 14 and Figure 15 show the local registration effect using the 3 methods considered.

Figure 14 and Figure 15 show the local image with 3 methods to compare the effectiveness of the registration. Our method has obvious optimization effects compared with method I and approaches the effect of method III. Moreover, our method is completed without manual intervention, unlike method III. For quantitative analysis, Table 3 lists the imaging position and angle correction value of a sequence of panoramic/fish-eye images using our method, Table 4 lists the registration accuracy of a sequence of panoramic/fish-eye images for the 3 methods considered.

**Figure 16:**
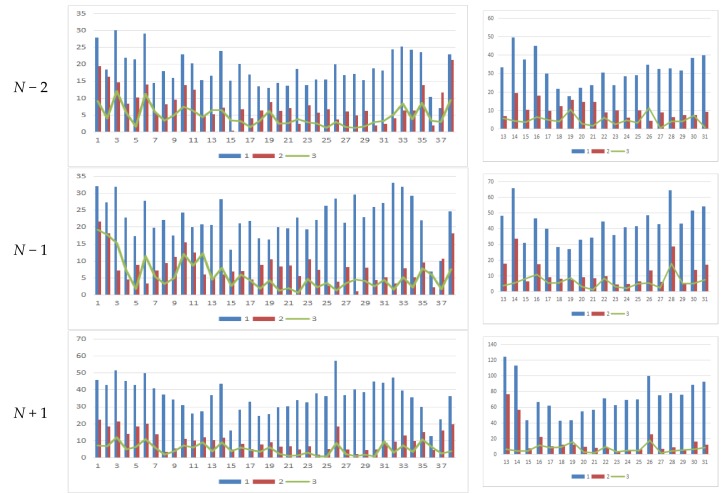

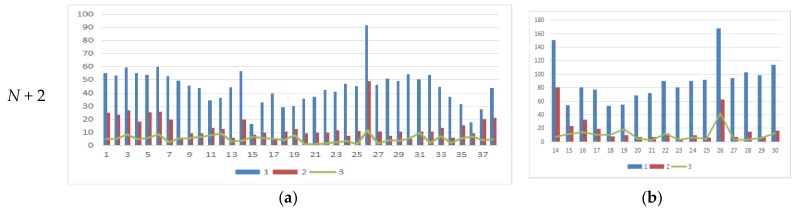
The error of 38/19(17) control points in the panoramic/fish-eye image sequence with 3 methods, series 1–3 expresses the error of each control point in method I, II and III, respectively. (**a**) Panoramic image(*No.*1–38); (**b**) Fish-eye image(*No.*13(14)–31(30)).

As shown in Table 3, the interval between sequence images is approximately 7 m, and the road height difference is within 0.3 m. The angle correction values of each panoramic/fish-eye image are different. Although the difference is small in most cases, it must be solved separately, rather than used directly for the next image. Table 4 shows that the registration error of method III is the smallest; the error of method II is approximately 2 times that of method III; and the error of method I is approximately 3 times that of method II. For further analysis, as shown in Figure 16, the position error of each control point using the 3 methods in a sequence of images is analyzed in detail.

As shown in Figure 16a,b, the error of each control point is noticeably reduced compared with that of method I. Moreover, the error distribution of the control point has a certain regularity with our method. First, the error of control points located at both ends of a panoramic image is large, such as *no.* (1, 2, 37 and 38). Second, the control points located at the top of the image have a larger error, such as *no.* (14 and 26) in the panoramic/fish-eye image (*N* + 1, *N* + 2), leading to a greater registration error of the fish-eye image (*N* + 1, *N* + 2) and the panoramic image (*N* + 2) than that of the others.

## 4. Discussion

### 4.1. Parameters in Skyline Pixels/Points Mat Ching

The parameters in skyline matching are angle error range (*w*), parameter segmentation (*t*) and gross error threshold (*e*), and keep the same in panoramic/fish-eye image registration. *w* is the iteration range of angle initial values (*r_x_ r_y_ r_z_*), in this paper, which is obtained from IMU. If IMU is unavailable, the approximate values of *r_x_*, *r_z_* could be solved by adjacent imaging position, and *r_y_* is set to 0. *t* determines the efficiency of registration. The nodes are set evenly in the correction angles (*r_x_*′ *r_y_*′ *r_z_*′), and it produces (*t* + 1)^3^ images at each iteration. The number of iterations can be reduced when *t* is large, but the amount of computation will be increased by power function. In order to ensure the accuracy of registration, *w* is reduced to half not *w*/*t* after each iteration. *e* is the key parameter of optimization; the matching skyline pixels/points less than *e* are considered as successful matches. When *e* is larger, it would be more mismatched points, but when *e* is small, it would filter out correct matching pixels/points. In addition, *e* is related to the image’s rows, the rows of the panoramic and fish-eye images is 4000 and 6000 pixels respectively in our experiments, it is empirically proved that setting *e* to 5 pixels is suitable.

### 4.2. The Precision and Automation Compare with Other Methods

Our method does not require manual intervention in the registration of panoramic/fish-eye images and LiDAR points. In recent studies, some articles use primitive pairs as line and plane, and a rigid registration to obtain high-precision result. However, extracting primitive pairs automatically from images and LiDAR is still challenging. Cui et al. [1] extracted line features from LiDAR points automatically and from the corresponding rectified mono-images through a semi-automation process, and achieved the registration accuracy of 4.718 and 4.244 pixels (image size is 1616 × 1232 pixels) in spherical and panoramic camera respectively. However, the process requires a manual check for primitive pairs before registration. Li et al. [2] took parked vehicles as registration primitives, and extracted them from panoramic images via Faster-RCNN; particle swarm optimization was further utilized to refine the translations between the panoramic camera and the laser scanner and registration error less than 3 pixels (image size is 2048 × 4096 pixels) are obtained. However, this automatic registration method relies on the parked vehicles, which makes its application scenarios limited. Moreover, the extraction of vehicles often fails due to poor illumination or occlusion. Our method takes skyline as primitive pairs as it is easy to extract both from image and LiDAR and skyline exists in most of scenes. In addition, our method can be used for both fish-eye image and panoramic image.

### 4.3. Weakness and Future Work

The main weakness of our method is the dependence on the skyline. In an open area, the image pixels of skyline could represent very distant objects, while points cloud are limited in long distance, making skyline pixels/points failed to match. Therefore, our method is more suitable for urban area or similar places with nearby skyline. There are also some problems require further study, such as the optimization of the position parameters which could be iterated alternately with the attitude parameters. The stitching error of the panoramic image also needs to be further studied as the camera center of each fish-eye lens hardly overlaps, and the attitude of each lens is seldom in the same horizontal plane.

## 5. Conclusions

In this paper, we proposed a skyline-based method for automatic registration of LiDAR points and panoramic/fish-eye image sequence in an MMS. The effectiveness of this method was demonstrated by comparison with the original registration and control points registration methods. Compared with other related works, the main contribution of this study is that (1) The panoramic/fish-eye image registration model is efficient and can achieve high-precision registration of an image and LiDAR points in an MMS. (2) The skyline-based registration method can automatically optimize the initial attitude parameters and realize high-precision registration of panoramic/fish-eye images and LiDAR points in an MMS. (3) The attitude correction values are different in panoramic/fish-eye image sequence; each value must be solved individually.

## Figures and Tables

**Table 1:** Skyline registration error in each iteration (*w*, *r_x_*′, *r_y_*′, *r_z_*′(degrees), *δ*(pixels)).

*NO.*	*w*	Panoramic Image					Fish-Eye Image				
		*r_x_*′	*r_y_*′	*r_z_*′	*n*	*δ*	*r_x_*′	*r_y_*′	*r_z_*′	*n*	*δ*
1	5	3.333	0	0	1263	10.914	0	1.666	0	205	28.850
2	2.5	3.333	0	0	1263	10.914	0	0.833	0	320	23.250
3	1.25	3.75	−0.416	0	1293	11.035	0	1.25	−0.416	373	15.231
4	0.625	3.541	−0.208	0	1391	9.906	−0.208	1.041	−0.416	415	17.105
5	0.312	3.541	−0.208	0.104	1392	8.895	−0.104	1.041	−0.520	439	15.493
6	0.156	3.489	−0.104	0.156	1420	8.692	−0.104	1.093	−0.520	454	14.723

**Table 2:** Registration accuracy with 3 methods (pixels).

Methods	I	II	III
Panoramic image (4000 × 8000)	29.041	8.692	5.883
Fish-eye image (6000 × 4000)	56.347	14.723	4.792

**Table 3:** Registration parameters of panoramic/fish-eye image sequence (*X_S_*,*Y_S_*,*Z_S_*(meters), *r_x_*′, *r_y_*′, *r_z_*′(degrees)).

*NO.*	Imaging Position			Panoramic Image			Fish-Eye Image		
	*X_S_*	*Y_S_*	*Z_S_*	*r_x_*′	*r_y_*′	*r_z_*′	*r_x_*′	*r_y_*′	*r_z_*′
*N* − 2	710.416	714.012	12.220	3.229	0.104	−0.104	0	0.416	−0.520
*N* − 1	705.175	708.426	12.249	3.333	−0.208	0.208	−0.104	0.729	−0.416
*N*	699.901	702.818	12.294	3.489	−0.104	0.156	−0.104	1.093	−0.520
*N* + 1	694.606	697.180	12.376	3.958	−0.104	0.208	−0.208	1.458	−0.520
*N* + 2	689.282	691.499	12.494	4.375	−0.104	0	−0.104	2.083	−0.416

**Table 4:** Registration accuracy of panoramic/fish-eye image sequence with 3 methods (pixels).

Methods	Panoramic Image				Fish-Eye Image			
	*N* − 2	*N* − 1	*N* + 1	*N* + 2	*N* − 2	*N* − 1	*N* + 1	*N* + 2
I	19.411	23.520	37.302	46.543	32.744	44.569	76.456	95.497
II	9.305	9.199	11.772	16.439	11.310	14.306	24.651	28.169
III	5.342	7.204	5.674	5.336	5.264	6.860	7.775	13.287

## Appendix A

**Table A1:** 3D coordinates of the control points (*m* = 38) (meters).

*No.*	1	2	3	4	5	6	7	8	9	10
*X*	736.872	750.315	720.542	720.324	720.490	720.626	725.968	738.342	736.746	729.746
*Y*	719.029	719.590	700.018	700.155	698.666	698.618	694.241	677.968	676.344	669.746
*Z*	21.071	18.655	13.994	12.090	12.129	14.009	18.400	23.493	23.547	21.818
*No.*	11	12	13	14	15	16	17	18	19	20
*X*	726.453	701.737	694.998	684.251	674.913	675.536	671.280	657.947	657.668	647.327
*Y*	664.691	668.587	675.141	639.304	614.069	615.144	649.908	626.820	626.328	624.664
*Z*	21.959	18.065	21.664	47.039	23.763	30.859	22.581	23.723	30.674	23.370
*No.*	21	22	23	24	25	26	27	28	29	30
*X*	623.430	609.152	632.710	657.169	625.455	680.693	601.633	649.663	626.746	651.056
*Y*	599.102	611.827	637.231	663.092	643.725	688.327	630.632	669.189	655.939	681.286
*Z*	24.280	24.345	23.444	22.564	17.430	21.746	21.645	17.741	21.697	21.765
*No.*	31	32	33	34	35	36	37	38		
*X*	673.819	650.928	657.382	656.167	678.039	686.893	707.353	722.722		
*Y*	695.005	704.462	711.380	720.494	743.051	745.828	741.755	730.199		
*Z*	18.171	30.328	30.201	35.273	35.389	30.197	22.362	17.831		

**Table A2:** 2D coordinates of control points in the panoramic image (*m* = 38) (pixels).

*No.*	*N* − 2		*N* − 1		*N*		*N* + 1		*N* + 2	
	*r*	*c*	*r*	*c*	*r*	*c*	*r*	*c*	*r*	*c*
1	762.4	1532.3	573.0	1602.9	458.6	1654.4	372.6	1686.2	311.4	1710.0
2	816.0	1743.5	675.4	1761.5	578.5	1779.7	495.7	1788.2	431.4	1795.2
3	2223.2	1875.2	1638.1	1841.8	1167.7	1841.4	841.8	1846.6	638.3	1855.2
4	2218.3	2021.4	1629.8	1989.0	1161.4	1961.4	836.6	1944.6	636.6	1932.4
5	2270.4	2025.4	1715.5	1995.4	1243.4	1966.9	906.4	1948.5	688.6	1935.7
6	2274.7	1886.8	1721.9	1854.6	1249.9	1846.4	911.6	1850.2	693.6	1855.8
7	2168.3	1708.3	1772.0	1682.4	1412.3	1685.9	1116.3	1706.5	897.1	1728.1
8	2181.5	1709.2	1963.9	1690.5	1747.7	1681.3	1543.0	1681.6	1359.8	1686.8
9	2241.4	1715.1	2024.5	1694.6	1808.4	1682.8	1600.2	1681.0	1409.8	1685.7
10	2496.0	1788.7	2298.2	1763.6	2084.0	1743.9	1864.4	1733.0	1649.0	1724.7
11	2615.3	1808.2	2436.6	1781.4	2241.3	1761.9	2029.1	1745.7	1810.6	1735.8
12	3243.9	1903.1	3115.1	1876.4	2945.2	1842.4	2708.4	1807.7	2394.5	1764.2
13	3483.9	1784.3	3380.4	1729.2	3240.7	1653.4	3006.9	1552.0	2619.5	1417.9
14	3443.5	1534.8	3388.3	1489.2	3328.7	1439.4	3254.7	1388.0	3163.3	1331.7
15	3438.0	1926.4	3388.2	1917.7	3344.9	1907.0	3292.9	1903.4	3232.1	1893.7
16	3437.4	1845.8	3390.5	1830.9	3347.5	1816.2	3296.3	1804.9	3236.8	1788.8
17	3697.7	1896.4	3663.8	1880.7	3631.9	1860.7	3585.2	1843.0	3527.1	1813.4
18	3685.9	1926.1	3657.9	1916.7	3634.8	1905.8	3603.3	1901.3	3567.8	1891.0
19	3684.4	1841.6	3657.9	1825.3	3635.2	1809.3	3604.6	1794.6	3569.9	1773.6
20	3778.8	1941.3	3759.1	1935.6	3748.3	1927.4	3727.5	1925.4	3707.0	1918.6
21	3819.7	1963.9	3806.1	1962.1	3803.2	1958.6	3790.1	1961.8	3779.9	1962.1
22	3993.6	1965.0	3987.4	1962.3	3992.1	1957.5	3993.0	1960.4	3994.3	1961.2
23	4001.0	1939.1	3998.2	1932.4	4009.4	1926.0	4011.1	1921.4	4015.8	1915.2
24	4023.7	1892.1	4026.1	1876.1	4042.1	1854.8	4053.8	1833.4	4069.9	1800.3
25	4114.3	2009.8	4125.5	2012.3	4139.7	2007.7	4154.0	2011.3	4171.4	2013.4
26	4089.7	1767.3	4117.3	1703.1	4174.5	1599.6	4275.7	1426.1	4543.3	1081.8
27	4162.1	1982.9	4168.9	1979.4	4188.0	1978.4	4201.5	1980.8	4216.9	1982.3
28	4186.4	1977.0	4212.6	1974.5	4243.9	1961.3	4284.7	1955.6	4338.2	1945.3
29	4221.8	1949.5	4237.7	1944.8	4267.1	1938.3	4296.2	1933.8	4330.0	1928.1
30	4355.6	1888.0	4400.3	1870.7	4467.2	1848.4	4549.5	1822.8	4654.9	1787.1
31	4387.3	1881.9	4479.4	1847.7	4627.0	1796.6	4858.6	1718.3	5267.2	1608.7
32	4789.0	1684.7	4892.0	1650.0	5025.9	1607.2	5187.3	1562.4	5388.5	1517.6
33	4928.8	1634.0	5064.0	1591.9	5237.6	1546.5	5439.3	1498.4	5679.7	1465.7
34	5132.5	1535.0	5281.1	1495.2	5461.0	1457.7	5656.5	1424.8	5870.1	1405.3
35	5895.4	1393.5	6106.1	1389.7	6317.1	1402.2	6507.1	1423.2	6672.7	1442.0
36	6159.8	1449.5	6384.6	1463.5	6591.1	1489.5	6764.0	1515.8	6908.5	1541.8
37	6828.7	1515.5	7051.2	1576.1	7210.8	1633.2	7324.1	1658.3	7409.5	1687.8
38	7806.8	1584.7	7839.0	1675.3	7863.6	1729.8	7873.6	1753.5	7884.5	1772.4

**Table A3:** 2D coordinates of control points in the fish-eye image (*m* = 19/17) (pixels).

*No.*	*N* − 2		*N* − 1		*N*		*N* + 1		*N* + 2	
	*r*	*c*	*r*	*c*	*r*	*c*	*r*	*c*	*r*	*c*
13	928.4	2523.9	725.9	2400.8	454.9	2222.2	48.6	1971.8	***	***
14	890.5	1980.3	799.2	1880.2	705.5	1772.6	589.1	1655.5	462.5	1504.1
15	824.0	2832.7	723.2	2808.7	633.4	2790.8	526.4	2781.8	410.8	2753.7
16	830.4	2652.9	733.5	2622.1	648.5	2584.8	544.2	2560.5	427.9	2520.0
17	1365.0	2770.9	1296.6	2736.2	1229.3	2693.3	1135.9	2650.9	1016.4	2587.6
18	1339.2	2833.5	1282.7	2813.0	1241.8	2793.7	1169.3	2777.6	1097.5	2754.8
19	1338.5	2652.5	1288.4	2617.5	1243.8	2578.7	1179.5	2548.3	1111.4	2502.5
20	1532.3	2866.6	1496.0	2852.5	1469.4	2837.0	1430.9	2831.6	1389.2	2818.7
21	1621.3	2916.8	1597.3	2910.1	1584.0	2904.4	1564.8	2909.7	1543.9	2911.5
22	1985.9	2915.4	1984.5	2910.9	1989.1	2904.4	2000.1	2910.9	2010.7	2912.6
23	2008.4	2861.6	2007.3	2848.7	2022.0	2833.0	2032.5	2826.0	2048.1	2812.0
24	2056.6	2762.5	2066.1	2727.3	2093.4	2683.4	2121.2	2635.1	2162.8	2565.0
25	2249.2	3012.4	2266.5	3009.9	2300.2	3007.4	2332.8	3015.4	2381.4	3020.8
26	2195.5	2493.2	2253.8	2356.2	2362.6	2137.8	2537.5	1778.9	2914.3	1038.7
27	2351.7	2954.4	2368.7	2948.6	2403.1	2944.8	2436.6	2953.0	2477.8	2954.5
28	2402.9	2940.1	2467.5	2935.5	2522.5	2908.1	2612.3	2893.8	2735.6	2870.2
29	2477.3	2885.8	2514.2	2871.6	2572.8	2858.0	2636.4	2848.8	2716.6	2835.1
30	2757.4	2750.6	2852.6	2711.5	2988.3	2660.8	3158.2	2602.9	3384.3	2520.5
31	2826.6	2736.6	3014.4	2661.3	3313.0	2542.7	3771.2	2357.3	***	***

*** express *No.*13 and 31 control points are not in the fish-eye image (*N*+2).
